# In Vitro Propagation Technology for the Endangered Aquatic Species *Nymphoides coronata*

**DOI:** 10.3390/plants13111508

**Published:** 2024-05-30

**Authors:** Fei Lin, Yong Kang, Yamei Li, Yuhua Guo, Wei Wang, Guangsui Yang, Junmei Yin, Fenling Tang, Mamdouh A. Eissa

**Affiliations:** 1 Tropical Crops Genetic Resources Institute, CATAS, Key Laboratory of Crop Gene Resources and Germplasm Enhancement in Southern China, Ministry of Agriculture and Rual Affairs, Key Laboratory of Tropical Crops Germplasm Resources Genetic Improvement and Innovation of Hainan Province, The Engineering Technology Research Center of Tropical Ornamental Plant Germplasm Innovation and Utilization, Haikou 571101, China; linfei198201@163.com (F.L.); jusky@foxmail.com (Y.K.); lym6137@163.com (Y.L.); guoyuhua01@126.com (Y.G.); 13976572870@163.com (G.Y.); 2 Haikou Experimental Station, Chinese Academy of Tropical Agricultural Sciences, Haikou 571101, China; mamdouh.eisa@aun.edu.eg; 3 Hainan Agriculture School, Haikou 571101, China; 13036096596@163.com; 4 National Key Laboratory for Tropical Crop Breeding, Sanya Research Institute, CATAS, Sanya 572024, China; 5 Department of Soils and Water, Faculty of Agriculture, Assiut University, Assiut 71526, Egypt

**Keywords:** tissue culture, Nymphoides, plant growth regulators, 6-BA, IAA, NAA

## Abstract

*Nymphoides coronata* is an endangered aquatic plant species with significant medicinal and ecological importance. To preserve *N. coronata* from going extinct, we need to provide seedlings and efficient multiplication techniques so that it can be extensively studied. This study aimed to identify the most suitable sterilization treatment, growth medium, and substrate for the cultivation and propagation of *N. coronata*. Ethanol sterilization, fungicide treatment, and sterile water washing were the most important sterilization steps. A combination of 6-benzylaminopurine (6-BA) and indoleacetic acid (IAA) was the most suitable medium for bud induction and shoot proliferation. The use of α-naphthaleneacetic acid (NAA) increased the rooting rate and rooting time compared to indole-3-butyric acid (IBA). Increasing the concentration of NAA from 0.5 to 1.0 mg/L increased the rooting rate from 78 to 100% and reduced the rooting time from 7 to 5 days. The survival rate of *N. coronata* seedlings was 100% in a mixture of red soil and sand (1:1, *w*/*w*). As a result, the procedure mentioned above could potentially be used to safely propagate this rare species on a large scale. These findings provide valuable insights into the optimal conditions for the successful cultivation and propagation of *N. coronata*, which can contribute to the conservation and sustainable use of this important rare plant species.

## 1. Introduction

*Nymphoides coronata* (Dunn) Chun ex Y.D. Zhou & G.W. Hu is a species of aquatic plant that belongs to the family *Menyanthaceae* [1]. *N. coronata* was first found in the northeast of Guangdong, China, in 1912 [2]. The plant populations in Guangdong, China’s northeast, went extinct due to the effects of human activity, habitat degradation, and environmental pollution; researchers had not discovered any live plants or specimens until recently, in 2012 [1]. *N. coronata* is a perennial species that grows in shallow water or on wet soil in marshes, ponds, and slow-moving streams. The plant reproduces both sexually through seeding and asexually through rhizome growth or branching [1]. The rediscovery of *N. coronata* by Zhou et al. [1] is significant for several reasons. It adds to the increasing evidence that many plant species that were previously believed to be extinct may actually still be found in the wild. *N. coronata* provides an opportunity for the further study and understanding of the ecology, genetics, and conservation of this important aquatic plant species [1,3]. The reappraisal of *N. coronata* is an important contribution to the field of botany, and it underscores the need for continued research and conservation efforts to protect the diversity and richness of plant life on our planet [1,3,4,5].

Plant tissue culture has the potential to produce many clonal seedlings in a short time throughout the year, aiding in plant multiplication [6,7]. Direct multiple shoot induction is a more dependable technique for clonal multiplication and a valuable way to produce plantlets from young or mature plants with a reduced risk of genetic instability than by the other regeneration pathways [6,8]. The in vitro process’s success depends on a number of variables, including plant growth regulators and medium composition [9]. The conventional means of multiplying/propagating *N. coronata* and other aquatic macrophytes are challenging due to their slow propagation and low seed germination rates [10]. Sexual reproduction through seed remains difficult, and propagation through new shoots from rhizomes and sucker division also has low reproduction rates [11,12], while grafting is intricate and time-consuming [13]. Currently, no research has documented a method for effectively regenerating *N. coronata*, a significant uncommon species. In order to properly propagate this valuable aquatic plant in large quantities, we thus investigated an effective methodology for in vitro plant regeneration using *N. coronata* shoot induction.

Contamination is a real problem that opposes the progress and development of tissue culture technology [14]. Various methods are employed to eradicate bacterial and fungal contamination, such as heat and light inactivation, the use of fungicides and antibiotics, and varying times of sterilization based on the tissue type [15,16]. Plant type influences the sterilizing technique selection [17,18,19]. In plant tissue cultures, shoot regeneration can be induced from tissue explants by treatment with phytohormones [20,21]. The use of plant growth regulators, such as 6-BA and IAA, can promote bud induction and shoot proliferation [20,22]. Auxins are well acknowledged to play a significant role in plant roots [23]. Numerous studies have demonstrated that the administration of auxins, such as IBA and NAA, increases the length and volume of roots and that auxin and auxin transport play a critical role in the formation of lateral roots [24,25].

Studying the phylogeny and biogeography of *Nymphoides* in China and across the world requires a detailed study of *N. coronata*. However, the conservation and propagation of *N. coronata* are challenging due to factors such as low seed germination rates, slow growth, and susceptibility to microbial contamination. When Zhou et al. [1] rediscovered *N. coronata*, a species that had been eliminated for a century, in 2012, there were no established procedures for the large-scale propagation of this rare endangered species. To address these challenges, this study aimed to develop an efficient tissue culture and rapid propagation technology for *N. coronata*. This study focused on various stages of the plant’s growth cycle, including the selection and preparation of explants, sterilization, and the optimization of the growth medium and plant growth regulators.

## 2. Results

### 2.1. Effect of Different Disinfectants and Disinfection Time on the Sterilization of N. coronata Explants

**Figure 1:**
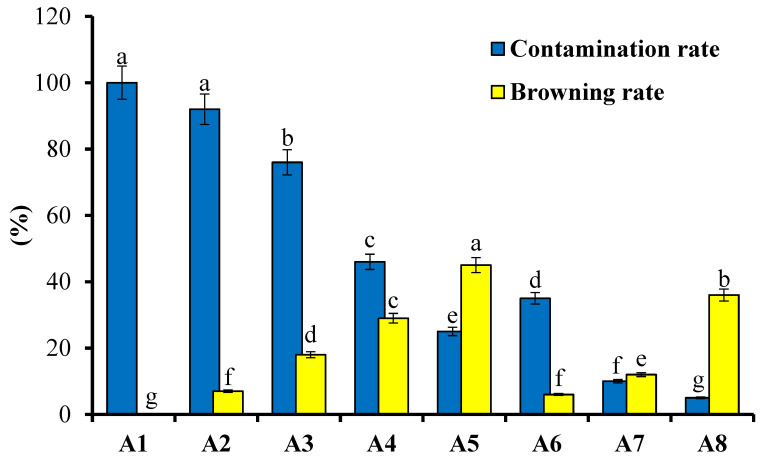
Effect of surface sterilization treatments for disinfection of *N. coronata* explants on contamination and browning rate. Means ± standard deviation with various letters refer to significant differences at *p* < 0.05. A1–A8 are the sterilization treatments as described in Table 1.

Different sterilization treatments (Table 1) were used for the disinfection of *N. coronata* explants, and the results of this study are shown in Figure 1. The contamination and browning rate (%) of the explants after being cultivated into the MS medium were used to test the efficiency of the studied sterilization procedures.

The efficacy of the surface sterilization treatments used on the *N. coronata* explants seemed to differ, based on the findings shown in Figure 1. The treatment A1, which used tap and sterile water as disinfectants, had a 100% contamination rate and was not effective in sterilizing the *N. coronata* explants. The use of Tween and mercuric chloride in sterilization treatments A3, A4, and A5 significantly reduced the contamination rate compared to A1 and A2. A5, which used 0.1% mercuric chloride with one drop of Tween for 20 min, significantly enhanced the sterilization effect and reduced the contamination rate. However, the browning rate of the explants increased significantly as the disinfection time increased in the case of 0.1% mercuric chloride. A8, which used 2% S206 fungicide for 90 min, had the best disinfection effect but also had a high browning rate. A7, which used 2% S206 fungicide for 60 min, had low contamination (10%) and browning (12%) rates and was the most suitable disinfection treatment based on comprehensive considerations of the disinfection effect, input, and environmental impact.

### 2.2. Effect of Different Plant Growth Regulators on Shoot Bud Induction of N. coronata

**Figure 2:**
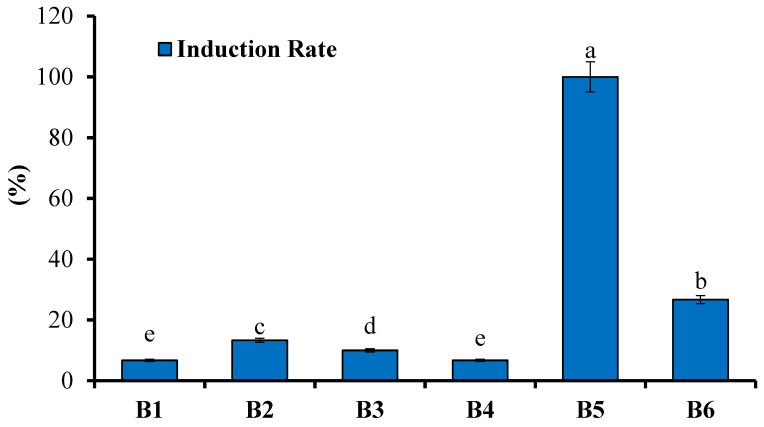
Effect of different plant growth regulators on the induction of shoot buds in *N. coronata* explants. Means ± standard deviation with various letters refer to significant difference at *p* < 0.05. B1–B6 are the growth regulators as described in Table 2.

As seen in Table 2 and Figure 2, IAA was found to be the most suitable plant growth regulator for inducing stem segments with buds in *N. coronata*, with a 100% induction rate, no callus formation, and a high number of clumped buds/adventitious shoots formation. While some adventitious buds were induced in the 2,4-D medium, there was more callus formation, making it more suitable for callogenesis. The NAA medium induced single buds, but these did not differentiate into adventitious buds. Similarly, in the IBA medium, there were single shoots, but these did not differentiate into clumped shoots.

### 2.3. Effect of Different Concentrations of 6-BA on the Proliferation of Clumped Shoots of N. coronata

**Figure 3:**
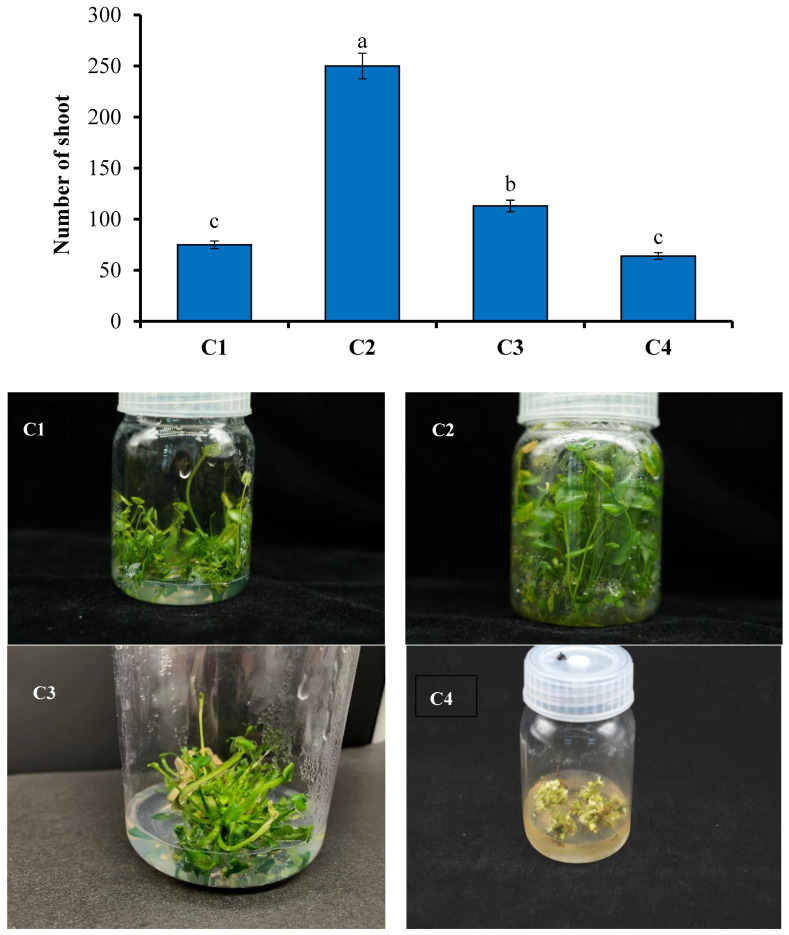
Effects of different concentrations of 6-BA on the proliferation of shoot clumps in *N. coronata*. Means ± standard deviation with various letters refer to significant differences at *p* < 0.05. (**C1**–**C4**) are the concentrations of 6-benzylaminopurine (6-BA) as described in Table 3.

Table 3 and Figure 3 demonstrate that the concentration of the plant growth regulator, i.e., 6-BA, had a significant impact on the multiplication of clustered shoots of *N. coronata*. As the concentration of 6-BA increased from 0.5 to 1.0 mg/L, the multiplication factor of clustered shoots also increased. However, above 1.0 mg/L, the formation of calluses also increased, which negatively affected bud differentiation and led to a decrease in the multiplication factor. The optimal concentration for proliferation was 1.0 mg/L of 6-BA, which resulted in a multiplication factor of 250. At this concentration, small cluster buds, stem segments, and leaves could be induced new cluster buds, which reproduced quickly and produced strong shoots.

### 2.4. Effects of Different Media on Rooting of Small Tufted Shoots of N. coronata

**Figure 4:**
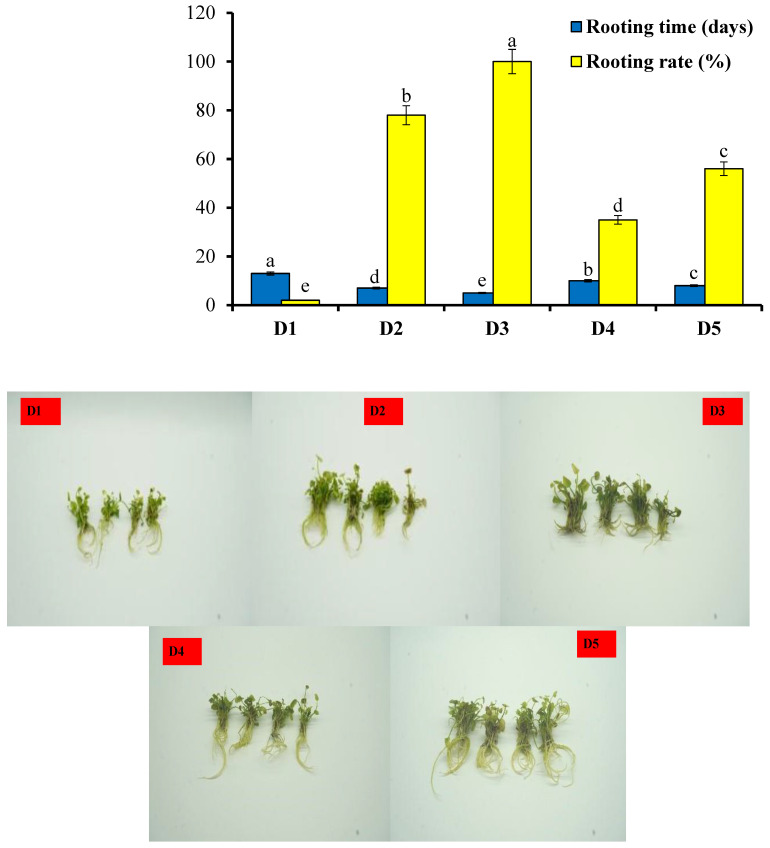
Effects of different concentrations of indole-3-butyric acid (IBA) and α-naphthaleneacetic acid (NAA) on the rooting of tissue-cultured *N. coronata*. Means ± standard deviation with various letters refer to significant differences at *p* < 0.05. (**D1**–**D5**) are concentrations of IBA and NAA as described in Table 4.

The use of different media caused significant differences in the rooting rate and rooting time (Figure 4). The earliest rooting time ranged between 5 and 13 days, while the rooting rate ranged between 35 and 100%. The results showed that the rooting rate of the tissue-cultured plantlets reached 100% in the treatment D3, followed by D2 with 78%. The lowest rooting rate was found in the D1 medium, which had the longest rooting time (13 days). The fastest rooting time was found in D3 (5 days), followed by D2 (7 days). According to the data in Table 4, the seedlings of *N. coronata* in D3 and D2 were strong with many long thick roots. The use of NAA in the growth media increased the rooting rate and rooting time compared to IBA. Moreover, increasing the concentration of NAA from 0.5 to 1.0 mg/L increased the rooting rate from 78 to 100% and reduced the rooting time from 7 to 5 days. 

### 2.5. Effect of Different Substrates on the Survival Rate of N. coronata Seedlings

**Figure 5:**
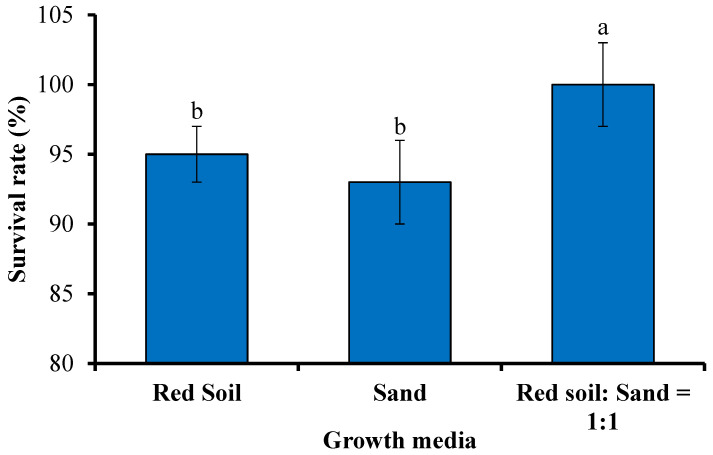
Effect of substrate on the survival rate of *N. coronata* seedlings. Means ± standard deviation with various letters refer to significant differences at *p* < 0.05.

Seedlings of *N. coronata* were cultivated in growth media, i.e., red soil, and sand, 1:1 ratio of red soil and sand, to study the effect of the substrate type on seedling growth (Figure 5 and Table 5). 

**Figure 6:**
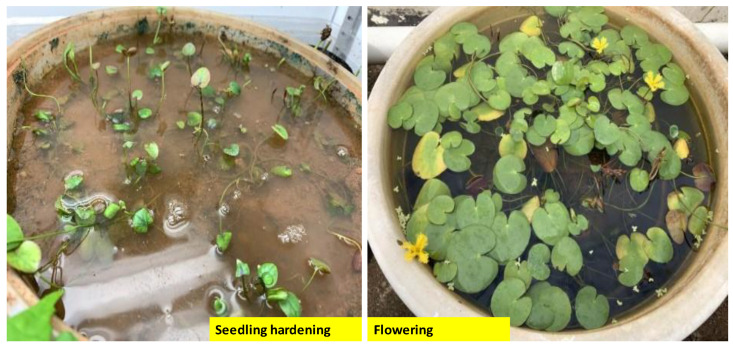
Hardening and flowering of *N. coronata* tissue cultured seedlings.

The results indicated that the survival rate of the *N. coronata* seedlings was high and not greatly affected by the type of refining substrate used. All three substrates tested yielded a survival rate over 93%, with the highest survival rate (100%) achieved in the substrate that comprised a mixture of red soil and sand in a ratio of 1:1. The seedlings were observed to be thick and strong and grew well in the mixture of red soil and sand. Flowering was also observed after 30 days of refining at a temperature of around 30 °C (Figure 6). These findings suggest that *N. coronata* seedlings are relatively easy to cultivate and can thrive in a variety of refining substrates, making them a promising species for further cultivation and propagation.

## 3. Discussion

The results of this study demonstrate that the use of a scientifically formulated rooting medium combined with appropriate sterilization and hormone treatments can result in high survival rates and healthy growth of *N. coronata* seedlings. The response of *N. coronata* to the sterilization treatments showed different effects when increasing the sterilization time. The sterilization treatment chosen is influenced by the plant type [17,18]. The most suitable sterilization treatment for explants involved a series of steps, including tap water rinsing, sterile water washing, ethanol sterilization, fungicide treatment, and sterile water washing. The best duration of soaking of the explants in the fungicide (2% S206) was 60 min. The use of this treatment resulted in a high survival rate of the explants, indicating that it is an effective way to sterilize explants and prevent contamination. This finding is consistent with the previous studies of Kowalik and Gródek [26]. In our tissue culture study, the fungicides aided in the restriction or eradication of contamination [26].

This study also identified the most suitable medium for the induction of buds and shoot proliferation, which are critical stages in the cultivation of *N. coronata*. The use of a combination of 6-benzylaminopurine (6-BA) and indoleacetic acid (IAA) was found to be the most suitable medium for bud induction and shoot proliferation. The induction rate of the buds reached 100% in B5, which contained 1 and 0.5 mg/L of 6-BA and IAA, respectively. In the abovementioned media, the shoot proliferation reached the maximum value (250). The high proliferation rate observed in this medium suggests that it can support the strong and healthy growth of *N. coronata* seedlings. These findings are consistent with previous studies that have shown that bud induction and shoot proliferation can be aided by the use of plant growth regulators, such as 6-BA and IAA [20,22]. The primary and most prevalent naturally occurring plant hormone, IAA, regulates nearly all aspects of plant growth and development, including senescence, cell division, elongation, and fruit production [20,21,27,28]. In tissue cultures, IAA is often used in combination with cytokinins to induce the formation of shoot buds from explants [20,21]. IAA promotes cell division and differentiation in explant tissue, which is necessary for the formation of new shoot buds. It also helps to establish polarity in the developing shoots, which is important for their proper growth and development [20,21,22]. In the current study, the optimum dose of 6-BA was 1.0 mg/L, and increasing the dose reduced the number of shoots. The precise role of 6-BA in shoot bud induction can depend on the plant species and the specific tissue culture conditions used [20,29,30]. In some cases, high levels of 6-BA can inhibit shoot bud formation, while in others, it may be required for optimal shoot regeneration [31,32]. The use of a combination of 6-BA and IAA was found to be the most suitable medium for bud induction and shoot proliferation of *N. coronata*. A combination of 6-BA and IAA in low concentrations has been found in several studies to enhance proliferation and regrowth in cultures [20,29,30].

The ideal auxin for *N. coronata* root development was determined by comparing different amounts of α-naphthaleneacetic acid (NAA) and indole-3-butyric acid (IBA). In terms of rooting, this study found that NAA was more suitable than IBA for the seedlings of *N. coronata*. The most suitable rooting medium was MS + 1.0 mg/L NAA + 1 g/L activated carbon, which resulted in a rooting rate of 100%. These findings suggest that appropriate hormone, i.e., NAA, treatments and rooting medium can contribute to the successful rooting and establishment of *N. coronata* seedlings. The superiority of NAA over other cytokinins was reported by Khan and Bi [23], Raju and Prasad [33], and Rostami and Movahedi [34]. The findings of Raju and Prasad [33] demonstrated that the kinds and amounts of hormones employed had a substantial impact on the rooting percentage. NAA is a synthetic cytokinin that has a similar structure to naturally occurring cytokinins, and it is widely used in plant tissue cultures to promote cell division and shoot bud induction [25]. Normally, NAA stimulates new cells by sending signals to the proteins, which causes the beginning and growth of lateral roots [35]. Various auxin hormones, including NAA, have the ability to regulate a plant’s growth and development. Previous studies confirmed that IBA and NAA cause roots to grow longer and larger, and auxin transport is essential for the development of lateral roots [24]. 

## 4. Materials and Methods

### 4.1. Plant Material and Explant Source

The materials, including *N. coronta* stem sections and disinfectants, were obtained from the Aquatic Plant Resource Garden of Haikou Experimental Station, Chinese Academy of Tropical Agricultural Sciences. The Aquatic Plant Resource Garden is a research facility dedicated to the collection, preservation, and study of aquatic plant species and is located in Haikou, Hainan, China. The stem sections were placed in a bucket or beaker with water during the transport to the laboratory and were processed immediately upon arrival. The initial processing involved the removal of any damaged or diseased tissue and the separation of the stem sections into suitable sizes for the experiment.

### 4.2. Sterilization and Inoculation Process for N. coronata Stem Sections

The 3–4 cm long nodal stem explants (stem sections with 1–2 buds) were first rinsed with water, then washed with detergent (1% of Tween 20), and rinsed with tap water for 30 min. The explants were then placed on a sterile operating table and sterilized with 75% alcohol for 1 min, followed by washing with sterile water three times. Eight different sterilization treatments were used, as shown in Table 1. After the sterilization treatments, the explants were rinsed with sterile distilled water five times and inoculated into half-strength MS medium. Each treatment was inoculated with 20 bottles, with one explant per bottle. The contamination rate was counted by incubating the bottles at 27 °C for 7 days and then calculating the infected plants. The contamination rate (%) was determined as the ratio of contaminated explants to the total number of explants inoculated multiplied by 100%. The browning rate (%) was calculated as the ratio of browning explants to the total number of explants inoculated, also multiplied by 100%. The browning rate (%) of the explants was also measured, with higher rates indicating a higher likelihood of tissue damage or death.

### 4.3. Culture Media

MS-based (Murashige and Skoog) growth media were used in this study. The pH of the medium was adjusted to 5.8, wherein 7 g/L carrageenan was added to the medium to make it solid, and it was autoclaved at 121 °C for 18 min. For shoot bud induction and shoot proliferation, the growth medium contained 30 g/L sucrose, while for rooting, it contained 15 g/L sucrose. 

### 4.4. Shoot Bud Induction and Shoot Proliferation

Half-strength solid MS medium (MS-based medium) was chosen as the basal culturing medium for shoot bud initiation [36,37]. The sterilized explants were cut off with a scalpel to remove the extra material containing any disinfectant. The sections were then placed into the solid medium with different phytohormones/plant growth regulators, as shown in Table 2. Samples of 6-benzyladenine (6-BA) alone (1.0 mg/L) and in combination with 0.5 mg/L each of α-naphthaleneacetic acid (NAA), indole-3-butyric acid (IBA), 3-indoleacetic acid (IAA), and 2,4-dichlorophenoxyacetic acid (2,4-D) were tested for the best combination of phytohormones. Twenty bottles, each containing three explants, were used for the inoculation of each treatment. The cultures were incubated for one week at 26 ± 12 °C and then transferred to white LED with light intensity of 1000 to 1200 lux (Sunlight Protection LED Lamp, Zhi Yi Optoelectronics, Guzhen, Zhongshan, China) for 12 h per day. The cultures were maintained at a temperature of 26 ± 1 °C for 20 days of incubation, after which the bud induction rate was recorded, and the growth status of the buds was noted. The induced buds were cut off and inserted into different adventitious shoot bud proliferation media, as shown in Table 3. Briefly, the media contained half-strength MS medium supplemented with a range of 6-BA concentrations (0.5, 1.0, 1.5, and 2.0 mg/L) and 0.5 mg/L IAA in order to standardize the maximum rate of shoot multiplication. Each medium was inoculated with 5 explants per bottle, and 20 bottles of each medium were used. The cultures were placed under white LED lighting for 12 h per day and maintained at a temperature of 26 ± 1 °C. The results were counted as the number of new buds/shoots per explant after 15–20 days.

### 4.5. Rooting of Strong Seedlings

The clumped seedlings were selected based on their consistent and robust growth. Individual buds/shoots or small clumps of seedlings (≥1.2 cm height) were then cut off and inoculated into different rooting media, as shown in Table 4. The rooting media contained half-strength MS medium supplemented with 1.0 g/L activated carbon and 0.5 or 1.0 mg/L of either NAA or IBA. Every medium was utilized in 20 bottles, each containing 5 seedlings for inoculation. The cultures were incubated under white LED lighting for 16 h per day and maintained at a temperature of 26 ± 1 °C. After 20 days of incubation, the rooting rate and the root initiation time were counted, and root growth state was noted.

### 4.6. Hardening and Transplanting

The rooted seedlings were moved to indirect sunlight for approximately one week and then slowly moved to a place with better light for refinement for about one week. The bottles containing the seedlings were turned often to ensure healthy growth. The root medium of the refined seedlings was washed out, and the seedlings were then placed in pots with different substrates, as shown in Table 5. The red soil was paddy soil with a pH value of 6.74 in 0.01 M CaCl_2_. Care was taken to pour water just over the plants, avoiding water that was too deep, which could affect seedling growth. Ten pots were used per treatment, with 10 plants per pot. The plants were placed under natural light, and the survival rate was counted after 20 days.

### 4.7. Data Analysis

Tukey’s test, a data analysis tool, was performed using SPSS version 18.0 (SPSS Inc., Chicago, IL, USA).

## 5. Conclusions

*N. coronata* was recorded for the first time in 1912 in Guangdong, China, and it then disappeared as a result of environmental pollution, habitat deterioration, and human activity, and it was then rediscovered again after 100 years in Hainan, China. The International Union for Conservation of Nature listed *N. coronata* as a highly endangered plant, and until 2012, scientists had not discovered any living specimens or plants of the species. The conservation and propagation of *N. coronata* plants can be challenging due to factors such as low seed germination rates and slow growth. This statement summarizes the findings of various tests related to the propagation of *N. coronata*. The current study included the most suitable sterilization treatment for explants, the most suitable medium for bud induction and seedling proliferation, the most suitable rooting hormone and medium, and the effect of different substrates on seedling growth. The findings suggest that ethanol sterilization and fungicide treatment are the most important sterilization steps for explants. Moreover, a combination of benzylaminopurine and indoleacetic acid is the most suitable medium for bud induction and seedling proliferation, while the use of α-naphthaleneacetic acid is suitable for rooting. This method will provide enough plants of the species that went extinct 100 years ago, which could be further used for research oriented to understanding the phylogeny and biogeography of *Nymphoides* in China and throughout the world.

## Figures and Tables

**Table 1:** Surface sterilization treatments for disinfection of *N. coronata* explants.

Treatment Abbreviation	Disinfection Treatment
A1	Tap water rinsing for 30 min → sterile water washing three times → sterile water washing five times and inoculation into MS medium.
A2	Tap water rinsing for 30 min → sterile water washing three times → sterilization with 75% ethanol for 30 s → sterile water washing five times and inoculation into MS medium.
A3	Tap water rinsing for 30 min → sterile water washing three times → sterilization with 75% ethanol for 30 s → sterile water washing five times → 0.1% mercuric chloride containing one drop of Tween for 10 min → sterile water washing five times and inoculation into MS medium.
A4	Tap water rinsing for 30 min → sterile water washing three times → sterilization with 75% ethanol for 30 s → sterile water washing five times → 0.1% mercuric chloride containing one drop of Tween for 15 min → sterile water washing five times and inoculation into MS medium.
A5	Tap water for 30 min → sterile water washing three times → sterilization with 75% ethanol for 30 s → sterile water washing three times → 0.1% mercuric chloride containing one drop of Tween for 20 min → sterile water washing five times and inoculation into MS medium.
A6	Tap water rinsing for 30 min → sterile water washing three times → sterilization with 75% ethanol for 30 s → sterile water washing five times → fungicide (2% S206) for 30 min → sterile water washing five times and inoculation into MS medium.
A7	Tap water rinsing for 30 min → sterile water washing three times → sterilization with 75% ethanol for 30 s → sterile water washing five times → fungicide (2% S206) for 60 min → sterile water washing five times and inoculation into MS medium.
A8	Tap water rinse for 30 min → sterile water washing three times → sterilization with 75% ethanol for 30 s → sterile water washing five times → fungicide (2% S206) for 90 min → sterile water washing five times and inoculation into MS medium.

**Table 2:** Effects of different plant growth regulators on the induction of shoot buds in *N. coronata* explants. Murashige and Skoog (MS) culture medium, 6-benzylaminopurine (6-BA), α-naphthaleneacetic acid (NAA), indole-3-butyric acid (IBA), indoleacetic acid (IAA), and 2,4-dichlorophenoxyacetic acid (2,4-D).

Medium Abbreviation	Shoot Bud Induction Medium	Bud Growth Status
B1	MS	No callus formation occurred, appearance of only small number of single buds with no adventitious shoots forming.
B2	MS + 6-BA 1.0 mg/L	Callus formation occurred, with a small number of single buds appearing, which could not be differentiated, and no adventitious shoots formed.
B3	MS + 6-BA 1.0 mg/L + NAA 0.5 mg/L	Callus formation occurred, with a small number of single buds appearing, but the buds were difficult to differentiate, and no adventitious shoots were formed.
B4	MS + 6-BA 1.0 mg/L + IBA 0.5 mg/L	Without callus formation, there were only a few individual single buds, and no adventitious shoots were formed.
B5	MS + 6-BA 1.0 mg/L + IAA 0.5 mg/L	Without callus formation, there was a large number of adventitious shoots, which grew vigorously.
B6	MS + 6-BA 1.0 mg/L + 2,4-D 0.5 mg/L	There was a large amount of callus formation and some adventitious shoots formed, which makes it suitable for use as a callus induction method.

**Table 3:** Effects of different concentrations of 6-benzylaminopurine (6-BA) on the proliferation of shoot clumps in *N. coronata*. Murashige and Skoog (MS) culture medium and indoleacetic acid (IAA).

Medium Abbreviation	Bud Induction Media	Bud Growth
C1	MS + 6-BA 0.5 mg/L + IAA 0.5 mg/L	There was no healing formation, but there was a large number of clumped buds, and the clumped buds were growing robustly.
C2	MS + 6-BA 1.0 mg/L + IAA 0.5 mg/L	There was no guaiac formation, but there was a large number of clumped buds, more clumped buds proliferated than C1, and the clumped buds grew robustly.
C3	MS + 6-BA 1.5 mg/L + IAA 0.5 mg/L	There was healing wound formation, there was clump formation, the clump buds were slowly glassy, and the buds grew averagely and slowly
C4	MS + 6-BA 2.0 mg/L + IAA 0.5 mg/L	There was a large number of healing wounds, which inhibited the formation of clump buds and made it difficult to form sprouts.

**Table 4:** Effects of different concentrations of indole-3-butyric acid (IBA) and α-naphthaleneacetic acid (NAA) on the rooting of tissue-cultured seedlings in *N. coronata* explants. Murashige and Skoog culture medium (MS).

Medium Abbreviation	Rooting Medium Formulations	Rooting of Tissue Culture Seedlings
D1	MS + activated carbon 1 g/L	The seedlings were strong with short thin roots. The roots were few, and the rooting time was long.
D2	MS + NAA 0.5 mg /L + activated carbon 1 g/L	The seedlings were strong with thick long roots. There were many roots, and the rooting time was short.
D3	MS + NAA 1.0 mg/L + activated carbon 1 g/L	The seedlings were strong with long thick roots. The roots were many, and the rooting time was short.
D4	MS + IBA 0.5 mg/L + activated carbon 1 g/L	The seedlings were average with short thin roots. The roots were few, and the rooting time was long. The seedlings turned yellow quickly.
D5	MS + IBA 1.0 mg/L + activated carbon 1 g/L	The seedlings were average short thin roots. The roots were few, and the rooting time was short. The seedlings and roots were prone to yellowing.

**Table 5:** Effect of substrate on the growth of *N. coronata* seedlings.

Soil Substrates and Ratios for Seedling Development	Seedling Growth
Red soil	Good water retention, easy soil compaction, good seedling growth.
Sand	Poor water retention, insufficient nutrition, weak seedlings, average growth.
Red soil/sand = 1:1	Good water retention, strong seedlings, and good growth.

## Data Availability

All the data are included in the article.
